# Searching for novel cerebrospinal fluid biomarkers of tau pathology in frontotemporal dementia: an elusive quest

**DOI:** 10.1136/jnnp-2018-319266

**Published:** 2019-04-13

**Authors:** Martha S Foiani, Claudia Cicognola, Natalia Ermann, Ione O C Woollacott, Carolin Heller, Amanda J Heslegrave, Ashvini Keshavan, Ross W Paterson, Keqiang Ye, Johannes Kornhuber, Nick C Fox, Jonathan M Schott, Jason D Warren, Piotr Lewczuk, Henrik Zetterberg, Kaj Blennow, Kina Höglund, Jonathan D Rohrer

**Affiliations:** 1 UK Dementia Research Institute, UCL Institute of Neurology, London, UK; 2 Department of Neurodegenerative Disease, UCL Institute of Neurology, London, UK; 3 Department of Psychiatry and Neurochemistry, Institute of Neuroscience and Physiology, The Sahlgrenska Academy at the University of Gothenburg, Gothenburg, Sweden; 4 Department of Psychiatry and Psychotherapy, Universitätsklinikum Erlangen and Friedrich‐Alexander Universität Erlangen‐Nuremberg, Erlangen, Germany; 5 Pathology & Laboratory Medicine, Experimental Pathology, Emory University School of Medicine, Atlanta, Georgia, USA; 6 Department of Neurodegeneration Diagnostics, Medical University of Bialystok, Bialystok, Poland; 7 Clinical Neurochemistry Laboratory, Sahlgrenska University Hospital, Mölndal, Sweden

**Keywords:** frontotemporal dementia, CSF, tau

## Abstract

**Background:**

Frontotemporal dementia (FTD) is a pathologically heterogeneous neurodegenerative disorder associated usually with tau or TDP-43 pathology, although some phenotypes such as logopenic variant primary progressive aphasia are more commonly associated with Alzheimer’s disease pathology. Currently, there are no biomarkers able to diagnose the underlying pathology during life. In this study, we aimed to investigate the potential of novel tau species within cerebrospinal fluid (CSF) as biomarkers for tau pathology in FTD.

**Methods:**

86 participants were included: 66 with a clinical diagnosis within the FTD spectrum and 20 healthy controls. Immunoassays targeting tau fragments N-123, N-mid-region, N-224 and X-368, as well as a non-phosphorylated form of tau were measured in CSF, along with total-tau (T-tau) and phospho-tau (P-tau_(181)_). Patients with FTD were grouped based on their Aβ_42_ level into those likely to have underlying Alzheimer’s disease (AD) pathology (n=21) and those with likely frontotemporal lobar degeneration (FTLD) pathology (n=45). The FTLD group was then subgrouped based on their underlying clinical and genetic diagnoses into those with likely tau (n=7) or TDP-43 (n=18) pathology.

**Results:**

Significantly higher concentrations of tau N-mid-region, tau N-224 and non-phosphorylated tau were seen in both the AD group and FTLD group compared with controls. However, none of the novel tau species showed a significant difference between the AD and FTLD groups, nor between the TDP-43 and tau pathology groups. In a subanalysis, normalising for total-tau, none of the novel tau species provided a higher sensitivity and specificity to distinguish between tau and TDP-43 pathology than P-tau_(181)_/T-tau, which itself only had a sensitivity of 61.1% and specificity of 85.7% with a cut-off of <0.109.

**Conclusions:**

Despite investigating multiple novel CSF tau fragments, none show promise as an FTD biomarker and so the quest for in vivo markers of FTLD-tau pathology continues.

## Introduction

Frontotemporal dementia (FTD) is a common form of early-onset dementia, but it is pathologically heterogeneous, which precludes accurate diagnosis during life of the underlying molecular cause.^1^ The majority of patients with FTD have either tau or TDP-43 inclusions at post mortem, but at present there are no biomarkers that can reliably separate these groups from each other or from healthy controls. Currently available cerebrospinal fluid (CSF) measures of tau do not seem to represent the burden of cerebral tau pathology and are variably affected in different forms of FTD. Furthermore, they can be abnormal in other proteinopathies.^2–5^ However, studies indicate that the tau protein can be cleaved into multiple different fragments, which are actively secreted from cells and can therefore potentially be identified in CSF.^6 7^ In this study, we assessed the potential of novel CSF measures of different tau species as candidate biomarkers for FTD.

## Methods

### Participants

86 consecutively recruited participants with available CSF from the University College London FTD cohort studies were included in the study: 66 patients and 20 healthy cognitively normal controls. The 66 patients met consensus diagnostic criteria for either behavioural variant FTD (bvFTD) (21, of whom one patient had associated motor neuron disease)^8^ or primary progressive aphasia (PPA)^9^ (45). In the PPA cohort, 11 had semantic variant, 16 had non-fluent variant (of whom two patients had associated progressive supranuclear palsy), 15 had logopenic variant and 3 did not meet criteria for any of the three variants, named PPA-not otherwise specified (PPA-NOS).^9^ All patients were screened for mutations causative of FTD and 10 patients were found to have a pathogenic mutation: *MAPT* (4), *GRN* (3) and *C9orf72* (3).

### Measurement of CSF markers

CSF was collected, processed and stored at −80°C according to standardised procedures.^10 11^ Initially, the concentrations of the currently available markers of CSF T-tau, P-tau_(181)_ and Aβ_42_ were determined using sandwich ELISAs (INNOTEST; Fujirebio Europe N.V., Gent, Belgium) following manufacturer’s instructions.

**Figure 1:**
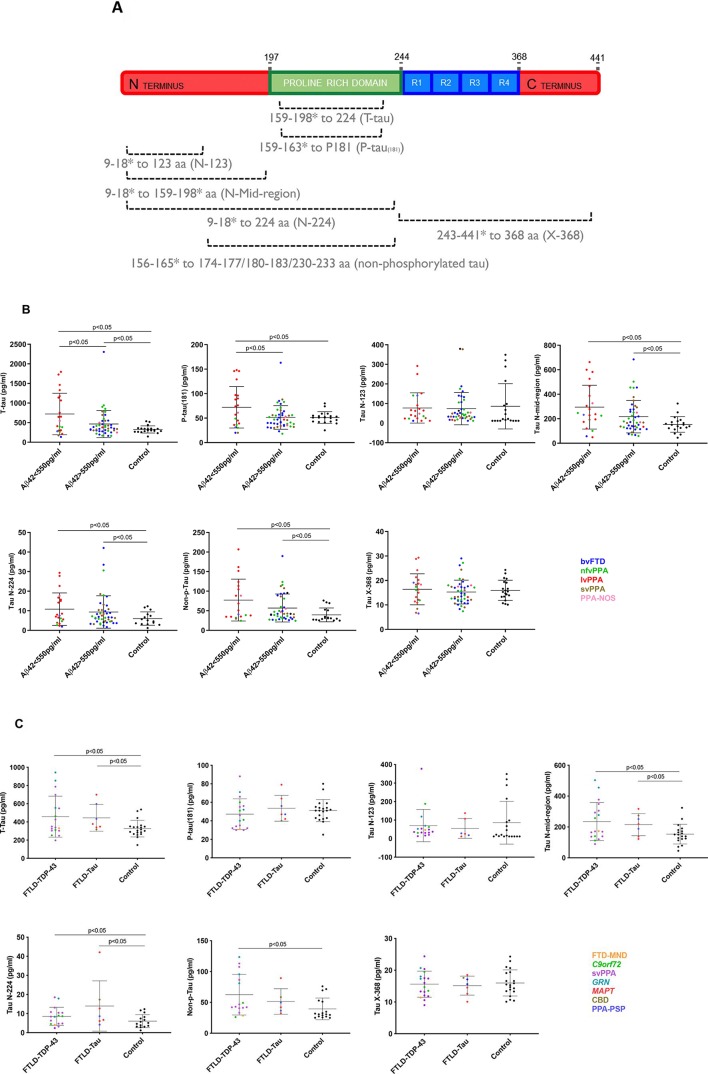

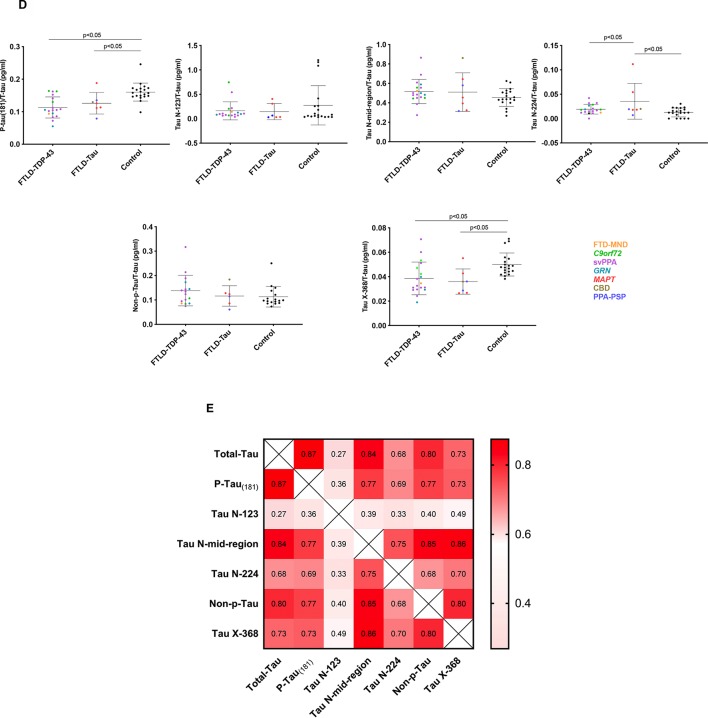
(A) Schematic of tau 441 aa protein with the approximate location of various linear epitope antibodies in the different immunoassays. Innotest T-tau uses AT120 (224 aa) as the capture antibody and a combination of HT7 (159–163 aa) and BT2 (194–198 aa) for detection. P-tau_(181)_ uses HT7 to capture the protein and AT270 (phosphorylated 181 aa) as detection. N-123 and N-224 use in-house developed Tau N-123 (123 aa) and Tau N-224 (224 aa) as capture antibodies, respectively, and Tau 12 (9 aa) as detection. N-Mid-region captures tau using Tau 12 and detects it with a combination of HT7 and BT2. X-368 uses Tau 368 (368 aa) as a capture antibody and K9JA (243–441 aa) as detection. Nonphosphorylated tau uses 1G2 as a capture (binds to the non-phosphorylated peptide sequences KTTP (174–177 aa), KTTP (180–183 aa) and RTTP (230–233 aa)) and 7E5 (156–165 aa) as detection. *represents polyclonal antibodies, and consequently the dotted lines represent approximate epitope location. (B) Comparison of T-tau (pg/ml), P-tau_(181)_, Tau N-123, Tau N-mid-region, Tau N-224, Non-p-Tau and Tau X-368 between patients with Aβ_42_<550pg/ml and >550pg/ml and healthy controls. Horizontal bars show mean and standard deviation. Colours in graph: blue: behavioural variant FTD (bvFTD); green: nonfluent variant PPA (nfvPPA); red: logopenic variant PPA (lvPPA); brown: semantic variant PPA (svPPA); pink: patients with a not otherwise specified variant of PPA (PPA-NOS). (C) Comparison of T-tau (pg/ml), P-tau_(181)_, Tau N-123, Tau N-mid-region, Tau N-224, Non-p-Tau and Tau X-368 between patients with probable Tau and TDP-43 pathology and healthy controls. Colours in graph: yellow: FTDMND; green: C9orf72 expansion mutation carriers; purple: svPPA; teal: GRN mutation carriers; red: MAPT mutation carriers; brown: corticobasal degeneration (CBD); blue: PPA-PSP: patients with PPA and progressive supranuclear palsy. (D) Comparison of P-tau_(181)_/T-tau, Tau N-123/T-tau, Tau N-midregion/T-tau, Tau N-224/T-tau, Non-p-Tau/T-tau and Tau X-368/T-tau between patients with probable Tau and TDP-43 pathology and healthy controls. Colours in graph: yellow: FTD-MND; green: C9orf72 expansion mutation carriers; purple: svPPA; teal: GRN mutation carriers; red: MAPT mutation carriers; brown: corticobasal degeneration (CBD); blue: PPA-PSP: patients with PPA and progressive supranuclear palsy. (E) Spearman’s correlation matrix between markers in a double gradient scale (pink, r=0; red, r=1). The p-value was less than 0.05 for all group comparisons.

We then performed five further ELISAs, one previously reported to identify non-phosphorylated forms of tau (performed as in Lewczuk *et al* 12), and four novel assays (figure 1A; online supplementary material):

10.1136/jnnp-2018-319266.supp1 Supplementary data

- *Tau N-123 amino acid (aa):* Plates were coated and incubated overnight at +4°C with in-house antibody anti-tau 123. Titrated calibrators (123 recombinant tau fragment) and sample were co-incubated with biotinylated detection antibody Tau 12 (Nordic Biosite). For detection, enhanced streptavidin–horseradish peroxidase (HRP) complex was used.
- *Tau N-224aa:* Magnetic beads (Quanterix, Lexington, Massachusetts, USA) were conjugated with the capture antibody anti-Tau 224 according to bead supplier’s conjugation protocol. Prior to each run, Tau 224 recombinant protein calibrator was serially diluted and the biotin-labelled antibody Tau 12 (Nordic Biosite) was used for detection.
- *Tau X-368aa:* Magnetic beads (Quanterix) were conjugated with capture antibody anti-Tau368. Tau 1–368 recombinant protein was serially diluted and used as calibrator. As detection antibody, biotin-labelled K9JA (Sigma) was used.
- *Tau N-mid-region:* Tau12 (Nordic Biosite, binding region aa9–18) was used as coating antibody and, as for detection, a combination of biotinylated HT7 (Thermo Scientific, aa159–163) and BT2 (Thermo Scientific, aa194–198). For detection, enhanced streptavidin–HRP complex was used. Full-length recombinant Tau 441 2N4R (rPeptide) was used as calibrator.

### Statistical analysis

Concentrations of tau species were compared between groups using a linear regression model in STATA V.14 (StataCorp, College Station, Texas, USA) with 95% bias-corrected bootstrapped CIs with 1000 repetitions. There was no difference in age and gender between controls and each of the disease groups (all comparisons >0.05; Kruskal-Wallis test, age; Fisher’s exact test, gender). The optimal cut-off point for each tau marker to differentiate tau from TDP-43 pathology was identified by selecting the concentration that produced the highest Youden index (*J*=sensitivity+specificity–1) using GraphPad Prism V.7 (GraphPad Software, San Diego, California, USA). The area under the curve (AUC) was calculated for each comparison. Spearman’s correlation coefficient (ρ) was used to investigate the association between tau species.

## Results

### Comparison of those with likely AD versus FTLD pathology (figure 1B, table 1, online supplementary table)

**Figure 2:**
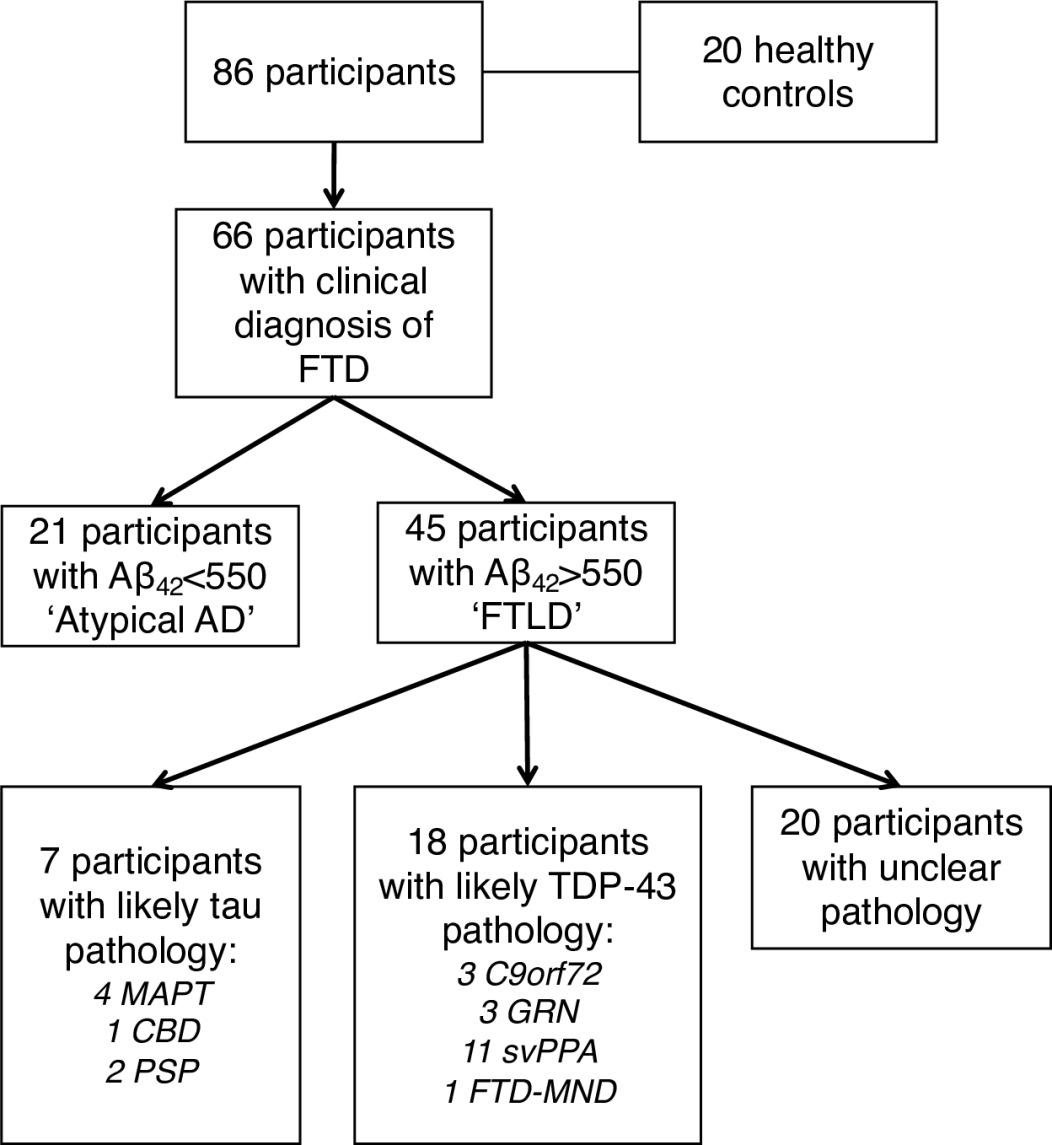
Flow diagram of participants included in the analysis. AD, Alzheimer’s disease; CBD, corticobasal degeneration; FTD, frontotemporal dementia; FTLD, frontotemporal lobar degeneration; PSP, progressive supranuclear palsy; svPPA, semantic variant primary progressive aphasia.

Some patients with bvFTD or PPA (particularly the logopenic variant) may have underlying Alzheimer’s disease (AD) pathology rather than frontotemporal lobar degeneration (FTLD) pathology. The initial analysis therefore aimed to compare these groups with controls. We used the Duits criteria^13^ to identify those patients who were likely to have underlying AD pathology (i.e. CSF concentrations of Aβ_42_ <550 pg/mL). This atypical AD group of 21 patients included 14 with logopenic variant PPA, 3 with non-fluent variant PPA, 1 with semantic variant PPA, 1 with PPA-NOS and 2 with bvFTD. Forty-five patients had Aβ_42_ >550 pg/mL and therefore were included in the FTLD group figure 2.

**Table 1:** Demographics and tau CSF marker concentrations for healthy controls, groups split by Duits criteria for likely AD pathology (vs FTLD pathology) and groups split by likely FTLD-tau and FTLD-TDP-43 pathology

	Healthy control	Aβ_42_<550 µL	Aβ_42_>550 µL	Probable tau pathology	Probable TDP-43 pathology
N	20	21	45	7	18
Male gender N (% group)	10 (50.0)	11 (52.3)	34 (75.5)	5 (71.4)	13 (72.2)
Age at CSF (years) Mean (SD)	64.2 (6.9)	65.8 (6.1)	64.1 (6.6)	64.8 (8.8)	62.3 (5.9)
T-tau pg/mL Mean (SD)	326.6 (90.8)	722.7 (529.1)	464.7 (344.1)	444.4 (146.4)	457.1 (224.6)
P-tau_(181)_ pg/mL Mean (SD)	51.1 (11.9)	72.1 (42.3)	51.3 (24.1)	53.5 (13.9)	47.2 (16.5)
Tau N-123 pg/mL Mean (SD)	85.8 (115.7)	77.4 (77.4)	74.0 (82.5)	54.9 (53.5)	70.6 (87.0)
Tau N-mid-region pg/mL Mean (SD)	153.4 (63.8)	294.7 (179.2)	216.7 (132.6)	215.4 (71.4)	235.1 (123.3)
Tau N-224 pg/mL Mean (SD)	6.0 (3.4)	10.8 (8.2)	9.3 (8.2)	13.9 (13.1)	8.5 (4.7)
Non-phosphorylated tau pg/mL Mean (SD)	39.4 (17.6)	77.2 (53.4)	57.0 (35.7)	51.4 (20.7)	62.5 (32.9)
Tau X-368 pg/mL Mean (SD)	16.0 (4.1)	16.3 (6.3)	15.2 (4.8)	15.1 (2.9)	15.6 (4.1)

AD, Alzheimer's disease; CSF, cerebrospinal fluid; FTD, frontotemporal dementia; FTLD, frontotemporal lobar degeneration.

The mean (SD) T-tau and P-tau_(181)_ concentrations were significantly higher in the AD group (722.7 (529.1) pg/mL; 72.1 (42.3) pg/mL) compared with healthy controls (326.6 (90.8) pg/mL; 51.1 (11.9) pg/mL) and the FTLD group (464.7 (344.1) pg/mL; 51.3 (24.1) pg/mL).

The AD group also showed significantly higher concentrations than controls for tau N-mid-region (AD, 294.7 (179.2) pg/mL; controls, 153.4 (63.8) pg/mL), tau N-224 (10.8 (8.2) pg/mL; 6.0 (3.4) pg/mL) and non-phosphorylated tau (77.2 (53.4) pg/mL; 39.4 (17.6) pg/mL). However, no significant differences were seen for tau N-123 (77.4 (77.4) pg/mL; 85.8 (115.7) pg/mL) or tau X-368 (16.3 (6.3) pg/mL; 16.0 (4.1) pg/mL).

The FTLD group showed significantly higher concentrations than controls for N-mid-region (216.7 (132.6) pg/mL), tau N-224 (9.3 (8.2) pg/mL) and non-phosphorylated tau (57.0 (35.7) pg/mL) but not for other novel measures.

None of the novel measures showed a significant difference between the AD and FTLD group.

### Comparison of those with likely FTLD-tau versus FTLD-TDP-43 pathology (figures 1C and D; table 1, online supplementary table)

Individuals in the FTLD group were then grouped based on their likely underlying pathology into an FTLD-tau group (containing *MAPT* mutation carriers, those with a secondary clinical diagnosis of progressive supranuclear palsy, and one patient with bvFTD who had subsequently come to post mortem and was found to have corticobasal degeneration; n=7) and an FTLD-TDP-43 group (containing *GRN* and *C9orf72* mutation carriers, those with a primary clinical diagnosis of semantic variant PPA or a secondary diagnosis of motor neuron disease; n=18) (figure 2).

CSF T-tau concentrations were significantly higher in the FTLD-TDP-43 group (457.1 (224.6) pg/mL) compared with controls, and also in the FTLD-tau group (444.4 (146.4) pg/mL) compared with controls.

Tau N-mid-region (235.1 (123.3) pg/mL), tau N-224 (8.5 (4.7) pg/mL) and non-phosphorylated tau (62.5 (32.9) pg/mL) also showed higher concentrations in the FTLD-TDP-43 group compared with healthy controls, while only concentrations of tau N-mid-region (215.4 (71.4) pg/mL) and tau N-224 (13.9 (13.1) pg/mL) were significantly higher in the FTLD-tau group compared with controls. No significant differences were seen for tau N-123 and tau X-368.

None of the measures showed a significant difference between the FTLD-TDP-43 and FTLD-tau groups.

We performed a subanalysis normalising tau markers for T-tau, based on previous literature which has shown an improved differentiation of tau and TDP-43 pathology using the ratio of P-tau_(181)_ to T-tau.^3–5^ The P-tau_(181)_/T-tau ratio was significantly lower for both the FTLD-TDP-43 group (mean (SD) 0.113 (0.032)) and the FTLD-tau group (0.126 (0.033)) compared with controls (0.160 (0.027)), but there was no significant difference between the FTLD-tau and FTLD-TDP-43 groups. Receiver operating characteristic (ROC) curve analysis measuring the ability of P-tau_(181)_/T-tau to differentiate probable FTLD-tau from FTLD-TDP-43 showed a sensitivity of 61.1% and specificity of 85.7% with a cut-off point of <0.109 and an AUC 0.63.

Of the novel tau species, both tau X-368 and tau N-224 had a significantly different ratio in the FTLD-tau group (mean (SD): 0.036 (0.010); 0.035 (0.037)) compared with controls (0.050 (0.009); 0.013 (0.009)). For tau X-368, the ratio was also lower in the FTLD-TDP-43 group (0.039 (0.013)) compared with controls, but there was no difference between the FTLD-tau and FTLD-TDP-43 groups. For tau N-224, there was also a significantly higher ratio for FTLD-tau compared with the FTLD-TDP-43 group (0.019 (0.010)). However, sensitivity and specificity of tau N-224 ratio to differentiate between likely FTLD-tau and FTLD-TDP-43 groups was only 61.1% and 57.1% with a cut-off point of <0.019 (AUC of 0.63). No significant differences were shown in the other novel tau measures.

### Correlations of tau CSF biomarkers

All CSF tau markers were significantly correlated with each other figure 1E. However, the strongest correlations were for T-tau with P-tau_(181)_ (ρ=0.87) and tau N-mid-region (ρ=0.84), and for tau N-mid-region with tau X-368 (ρ=0.86) and non-phosphorylated tau (ρ=0.85). Although significant, the correlations of Tau N-123 with the other tau species were fairly weak.

## Discussion

In this study, we investigated the potential of novel CSF tau measures as biomarkers of tau pathology in FTD. However, no significant differences were seen between those with likely underlying AD pathology and FTLD pathology, or between those with likely FTLD-tau and FTLD-TDP-43 pathology.

Tau N-224 was one of only two markers higher in the FTLD-tau group compared with controls, and when normalised for total-tau, showed a significant difference between FTLD-tau and FTLD-TDP-43, but separated the groups with only poor sensitivity and specificity of <65% (AUC of 0.63). A similar sensitivity (61.1%) and higher specificity (85.7%) was found for the P-tau_(181)_/T-tau ratio at a cut-off point of <0.109 (AUC of 0.63), a marker previously described by other groups: Hu *et al* 3 found a sensitivity of 82% and specificity of 62% for the comparison of FTLD-tau and FTLD-TDP-43 with a cut-off point of <0.372 (AUC of 0.73), while Borroni *et al* 4 found 83% and 64% with a ratio of <0.136 (AUC of 0.87), and Meeter *et al* 5, 67% and 76% with a cut-off point of <0.121 (AUC of 0.73). However, such a sensitivity and specificity would have limited clinical use as it would still result in considerable overlap between groups.

Although the fragments we have measured do not show a diagnostic accuracy that is superior to the existing tau biomarkers, we find different patterns in the concentrations of the fragments between the pathological groups. This finding is in concordance with other studies which suggest that tau may be differentially processed and secreted in a regulated manner.^14^ We hypothesise that specific tau fragments may be generated and secreted in different tauopathies, and here we provide evidence that three tau fragments are significantly increased in CSF in FTD compared with controls. It is likely that there are other fragments of tau, not analysed in this study, that are more specific to FTD, and further work is required to identify these.

This study has a number of limitations. The majority of the patients did not have pathological confirmation of the cause of their illness, and in addition, although there is a relatively large number of cases for a study of a rare disorder like FTD, the individual numbers are small in each subgroup. There are also potentially limitations in the assay sensitivities for the novel tau fragments such that improvement in these may lead to a clearer difference between cases and controls that is not currently apparent.

In conclusion, while a number of these novel tau species show significantly higher concentrations in those with underlying AD pathology, they do not show any added benefit above current tau biomarkers and are not useful as biomarkers of tau pathology in FTD. Further work in the development of biomarkers of tau and TDP-43 in FTD is needed, particularly in light of potential disease-modifying tau therapies currently entering clinical trials.
