# Supplementary material for: Searching for novel cerebrospinal fluid biomarkers of tau pathology in frontotemporal dementia: an elusive quest
Source: J Neurol Neurosurg Psychiatry. 2019 Apr 13;90(7):740–6. doi: 10.1136/jnnp-2018-319266 (PMC6585261; doi:10.1136/jnnp-2018-319266)
Supplement: Supplementary data [file jnnp-2018-319266supp001.pdf]

Supplementary Table

Adjusted mean differences in group comparisons of tau CSF markers for a) likely AD pathology versus FTLD pathology, AD vs controls and FTLD versus controls, and b) likely FTLD-tau versus FTLD-TDP-43 pathology, FTLD-tau vs controls and FTLD-TDP-43 vs controls. Mean differences (CI = 95% confidence intervals). Significant results in bold.

a)

	**AD vs FTLD**	**AD vs control**	**FTLD vs control**
**T-tau**	**258.0 (44.1, 539.3)**	**396.1 (183.3, 649.4)**	**138.1 (62.7, 296.5)**
**P-Tau(181)**	**20.7 (2.7, 41.7)**	**20.9 (3.6, 41.7)**	0.2 (-10.9, 7.0)
**Tau 9-123**	3.4 (-47.4, 34.4)	-8.3 (-45.5, 75.2)	-11.3 (-37.8, 76.2)
**Tau N-mid-region**	77.9 (-163.1, 1.45)	**141.2 (70.0, 228.6)**	**63.3 (13.7, 109.3)**
**Tau N-224**	1.4 (-5.9, 2.5)	**4.8 (1.3, 8.6)**	**3.3 (0.7, 6.7)**
**Non-phosphorylated tau**	20.2 (-49.2, 1.0)	**37.8 (15.9, 65.9)**	**17.6 (4.8, 31.5)**
**Tau X-368**	1.1 (-4.1, 1.6)	0.3 (-3.6, 2.5)	-0.7 (-1.4, 2.9)

b)

	**FTLD-TDP-43 vs tau**	**FTLD-tau vs control**	**FTLD-TDP-43 vs control**
**T-tau**	12.7 (-152.1, 131.0)	**117.8 (30.1, 258.1)**	**130.5 (32.1, 243.6)**
**P-Tau(181)**	-6.3 (-4.4, 20.2)	2.4 (-15.9, 6.1)	-3.8 (-6.2, 11.9)
**Tau 9-123**	15.6 (-75.0, 32.5)	-30.8 (-29.3, 97.4)	-15.1 (-50.5, 77.7)
**Tau N-mid-region**	19.6 (-93.9, 53.0)	**62.0 (7.8, 125.3)**	**81.6 (26.2, 151.0)**
**Tau N-224**	-5.4 (-1.4, 19.0)	**7.9 (1.6, 22.5)**	**2.5 (0.1, 5.5)**
**Non-phosphorylated tau**	11.0 (-31.4, 11.3)	11.9 (-31.9, 1.7)	**23.0 (6.6, 42.0)**
**Tau X-368**	0.5 (-3.4, 2.2)	-0.8 (-1.8, 3.8)	-0.3 (-2.2, 2.8)

Supplementary material

Assay validation

For the N-123 assay, LLOQ and ULOQ were respectively 12.2 pg/mL and 12500 pg/mL, determined by analysing the deviation from the true value of each calibrator point. The CV% for the back calculated concentrations of the data from the calibrator curve was <20% at LLOQ and ULOQ. Intra- and inter-assay variability were measured over 3 runs (11 plates) and were, respectively, 7.2% and 18.6% for the high QC sample and 3.8% and 26.1% for the low QC sample. LOD was 10.3 pg/mL.

In the Simoa assay N-224 assay, LLOQ and ULOQ were respectively 2.5 pg/mL and 160 pg/mL. The CV% for the back calculated concentrations of the data from the calibrator curve was <20% at LLOQ and ULOQ. The LOD was 0.05 pg/mL. Intra-assay variability was 2.7 % for the low QC and 4.8 % for the high QC. Inter-assay variability was 3.6 % for the low QC and 1.5 % for the high QC. QC samples were run in two duplicates.

For the Simoa X-368 assay, the calibration range for the assay was determined between 4.88 and 625 pg/mL. The LOD was determined by analyzing 16 duplicates of the blank. The mean AEB value was 0.0675 and the standard deviation was 0.01158. Adding three SD resulted in an AEB of 0.1023, which corresponds to a LOD of 3.6 pg/mL. Calibration curve data from five assay runs were used to determine the ULOQ and LLOQ where the relative error of the back-calculated concentrations for the calibrators was plotted as a function of concentration. LLOQ was set to 4.88 pg/mL and ULOQ to 625 pg/mL. The intra-assay CV was < 7.9% and the inter-assay CV measured < 12.3%.

In the N-mid-region assay, LLOQ and ULOQ were respectively 33 pg/mL and 6250 pg/mL. Intra- and inter-assay variability were measured over 6 runs and were, respectively, 9.2% and 10.7% for the high QC sample and 13.6% and 21.4% for the low QC sample. LOD was 7 pg/mL.
